# Developmental peculiarities in placentae of ovine uniparental conceptuses

**DOI:** 10.1371/journal.pone.0188278

**Published:** 2017-11-30

**Authors:** Roberta Arena, Federica Zacchini, Paola Toschi, Luca Palazzese, Marta Czernik, Grażyna Ewa Ptak

**Affiliations:** 1 Department of Experimental Embryology, Institute of Genetics and Animal Breeding, Polish Academy of Sciences, Jastrzebiec, Poland; 2 Faculty of Veterinary Medicine, University of Teramo, Teramo, Italy; 3 Malopolska Centre of Biotechnology, Jagiellonian University, Krakow, Poland; University of Connecticut, UNITED STATES

## Abstract

Genomic imprinting is an epigenetic phenomenon regulating mono-allelic expression of genes depending on their parental origin. Defective genomic imprinting is involved in several placental disorders, such as intrauterine growth restriction and pre-eclampsia. Uniparental embryos, having maternal-only or paternal-only genomes (*parthenogenotes* [PAR] and *androgenotes* [AND], respectively), are useful models to study placentation. The aim of this work was to reveal the effect of parental genome (maternal and paternal) on placentation. To do this, uniparental (AND and PAR) and biparental (CTR) *in vitro* produced sheep embryos transferred to recipient females were collected at day 20 of pregnancy and their placentae were analyzed. qPCR analysis showed that imprinted genes (*H19*, *IGF2R* and *DLK1*) were expressed accordingly to their parental origin while the expression f DNA methyltransferases (*) was disregulated*, especially in PAR (P < 0.05). AND placentae were significantly hypomethylated compared to both PAR and CTR (P = 0.023). Chorion-allantoid of AND showed impaired development of vessels and reduced mRNA expression of vasculogenetic factors (*ANG2* P = 0.05; *VEGFR2* P< 0.001; *TIE2* P < 0.001). Morphologically, PAR placentae were characterized by abnormal structure of the trophoectodermal epithelium and reduced total number (P<0.03) of Trophoblastic Binucleate Cells. A reduced implantation rate of both classes of uniparental embryos (P<0.03) was also noted. Our results provide new insights into the characterization of uniparental embryos and demonstrate the complementary role of parental genomes for the correct establishment of pregnancy. Thus, our findings may suggest new targets to improve our understanding of the origin of imprinting-related placental dysfunction.

## Introduction

Genomic imprinting is an epigenetic process responsible for the mono-allelic (maternal-only or paternal-only) expression of specific genes, known as imprinted genes. The key role of genomic imprinting during development was discovered in mammals thanks to the construction of uniparental embryos by pronuclear transfer [1,2]. These experiments showed that gynogenetic (maternal diploid genome) and androgenetic (paternal diploid genome) embryos failed to develop to term and showed an unbalanced feto/placenta ratio. In particular, gynogenetic and androgenetic embryos were characterized, respectively, by poor or extensive development of placental tissues [3], thus suggesting that parental genomes play complementary roles in placentation. The involvement of imprinted genes in placental development and function (*i*.*e*., nutrient transfer, fetal growth control) has been further confirmed by more recent research [4–7]. For example, reduced expression of *IGF2*, a paternally expressed gene, has been associated with growth restriction and impaired nutrient allocation from the mother [6], while low levels of *H19*, a maternally expressed gene, have been related to increased placental weight and fetal overgrowth [8–11]. Imprinting dysregulation has been described is several placental disorders, such as intrauterine growth restriction and pre-eclampsia, or in pregnancies obtained by assisted reproduction technologies [12–15]. The aim of this work was to study the effects of parental genome (maternal and paternal, separately) on early placentation. The study was performed on sheep (*ovis aries*) model as it is a powerful model for human pregnancy, due to similarities in placental development and physiology [16,17]. In mouse [1,3], uniparental conceptuses die during post-implantation period, around 10.5 day post coitum (dpc, Carnegie stage 14). The present study was carried out on ovine conceptuses at 20 day of pregnancy, which corresponds to 10.5 dpc in mouse. Our results provide new insight in early placentation of parthenogenetic and androgenetic conceptuses, which contributes to better understand the etiology of imprinting-related placental diseases.

## Materials and methods

All chemicals, unless otherwise indicated, were obtained from Sigma Chemicals Co. (St. Louis, MO, USA).

### Ethics statement

All animal experiments were performed in accordance with DPR 27/1/1992 (Animal Protection Regulations of Italy) in concordance with European Community regulation 86/609 and were approved by CEISA (Inter-Institutional Ethics Committee for Animal Experimentation) Prot. 79/2013/CEISA Prog. 58. The permit n°: CEISA VI, Classe 8.1, Prot. 2823. Sheep were pre-anesthetized with Acetyl Promazine (Prequillan, Fatro, Ozzano dell’Emilia, Italy), 1 ml IM and anesthetized with sodium thiopental (10 mg/kg BW, Pentothal Sodium, Intervet srl, Milano, Italy). These treatments alleviate level of suffering to minimum. After surgery, animals were kept in warm and dry place, isolated from animals until recovery.

### Oocyte collection and *in vitro* maturation (IVM)

Oocyte collection and *in vitro* maturation was performed as previously described [18]. Briefly, sheep ovaries were collected from local slaughterhouse (OVIN.COM S.R.L. Pianella, Italy) and transferred to the laboratory within 1–2 hours. Oocytes were aspirated with 21 G needles in presence of TCM-199 medium (Gibco, Thermo Fisher Scientific, Milan, Italy) containing Hepes and Heparin. Then, all oocytes with an unexpanded cumulus and uniform cytoplasm were selected for *in vitro* maturation (IVM). Maturation was conducted in 4-well culture plates (Nunclon, Roskilde, Denmark) containing 0.4 ml of IVM medium and incubated in a humidified atmosphere of 5% CO_2_ in air at 39°C for 24 h.

### Production of parthenogenetic (PAR) embryos

MII oocytes were activated with a combined treatment of ionomycin and 6-(dimethylamino)-purine, in Synthetic Oviductal Fluid (SOF) medium, as previously described [19].

### Production of androgenetic (AND) embryos

Matured oocytes were enucleated and *in vitro* fertilized as previously described [19]. Briefly, MII oocytes were denudated and subsequently enucleated in Ca^2+^/Mg^2+^-free manipulation medium. IVF was carried out as described below, but with a higher sperm concentration (25×10^6^ sperm/ml).

### Production of in vitro fertilized (CTR) embryos

*In vitro* fertilized embryos were produced as previously described [18]. Briefly, matured oocytes were partially stripped of cumulus cells by repeated pipetting. Frozen semen (SemenItaly, Inseme Spa, Italy) was rapidly thawed at 37°C and washed twice by centrifugation at 500 g for 5 min in bicarbonate-buffered SOF with 4 mg/ml BSA. *In vitro* fertilization (IVF) was carried out in 50 μl drops of IVF medium, using 5×10^6^ sperm/ml and a maximum of 15 oocytes per drop, at 38.5°C in 5% CO_2_ for 20 h.

### In vitro culture of preimplantation embryos

All classes of embryos were transferred into 20 μl drops of SOF medium enriched with 1% (v:v) Basal Medium Eagle (BME) essential amino acids, 1% (v:v) Minimum Essential Medium (MEM), non-essential amino acids (Gibco), 1 mM glutamine and 8 mg/ml fatty acid-free BSA (SOFaa-BSA). Zygotes were cultured in a humidified atmosphere of 5% CO_2_, 7% O_2_ and 88% N_2_ at 38.5°C.Medium was changed at day 3 and day 5 (supplemented with glucose). Cleavage was assessed at day 1 and blastocyst formation was recorded at day 6.

### Animal treatment, embryo transfer, and sample recovery

#### Animal treatment and care

Sardinian ewes (n = 30) obtained from local breeders were housed in the authorized experimental farm from the Istituto Zooprofilattico Sperimentale Abruzzo e Molise, Loc. Gattia, Italy, feed and kept under the best sheep housing standards. The synchronization of sheep was achieved with Crono-gest sponges of 25 mg (Intervet, Milan, Italy). After 12 days, Crono-gest sponges were removed and estrous monitored for 48h. Six days after estrous, embryo transfer was performed. Ewes were fasted for 24h before surgery and then were pre-anesthetized with 1 ml IM Acetyl Promazine (Prequillan, Fatro, Ozzano dell’Emilia, Italy) and anesthetized with sodium thiopental (10 mg/kg BW, Pentothal Sodium, Intervet Srl, Milano, Italy). These treatments alleviate level of suffering to minimum. After surgery, animals were kept in warm and dry place, isolated from animals until recovery. Post-operatory suffering alleviation was induced by intramuscular injection of flumixin meglumine (Zoetis, Rome, Italy). Antibiotic treatment consisted of intramuscular injection of ampicillin (0.2 g/10 kg, Amplital Vet, Ceva SpA, Agrate Brianza, Italy) every 24 hours for 3 days.

#### Embryo transfer

In vitro produced (IVP) blastocysts (2–4) were surgically transferred to recipient sheep (n = 10 for CTR, n = 20 each for AND and PAR) by paramedian laparotomy 6 days after estrus. After the exposition of uterine horns, a smooth catheter was introduced into the lumen, and embryos were deposited. After surgery animals were recovered as describe above.

#### Sample recovery

Conceptuses were recovered by paramedian laparotomy at day 20 of pregnancy. Immediately after collection, conceptuses were transferred into warm phosphate-buffered saline solution with Ca^2+^/Mg^2+^ and observed under a stereomicroscope to assess their vitality by the presence of a heartbeat. Early placental tissues were snap frozen in liquid nitrogen or fixed in paraformaldehyde for subsequent analysis. At the end of experiments, animals were scarified according to the national regulation and European Directive 2010/63/EU.

### Histological analysis

Chorion-allantoid tissues (n≥ 4 samples/group) were fixed in 4% (w:v) paraformaldehyde and subsequently dehydrated into increasing ethanol solutions (5 minutes at each step) and cleared in xylene mixture. Finally, placentae were paraplast embedded. 5 μm sections were used for hematoxylin eosin staining. Images were taken using the Nikon Eclipse E600 microscope (Firenze, Italy).

Trophoblastic cells were divided into Trophoblastic Binucleate Cells (BNC) and uninucleate cells. Histological analysis was performed on ≥15 randomly selected fields/sample (X 100).

Vasculogenetic development was assessed by classifying placental vessels into four developmental stages: *stage I*–early vasculogenesis with formation of tight-junctional contacts between angioblasts; *stage II* early–early tube formation with dilation of intercellular clefts and creation of the lumen precursor; *stage II late*–development of perivascular cells resembling pericytes and hematopoietic stem cells, which pass into the early lumen; *stage III*–late vasculogenesis/angiogenesis, with establishment of a basal lamina separating the lumen and endothelial cells from the perivascular cells [20,21]. Analysis was performed on ≥40 fields/sample and on ≥4 samples/group (magnification X 40).

### Global DNA methylation analysis

Genomic DNA (gDNA) was extracted from chorion–allantoid tissues (≥4 samples/group) using Wizard Genomic DNA purification system (Promega, Milan, Italy), according to the manufacturer’s instructions. Then, global DNA methylation was assessed by quantifying 5-methylcytosine (5-mC) using a fluorescence-based immunoassay, MethylFlash Methylated DNA Quantification Kit (EpiGentek, Farmingdale, NY, USA) according to the manufacturer’s protocol.

### Gene expression analysis

Total RNA from placental tissues (≥4 samples/group) was extracted using an SV Total RNA Isolation System (Promega, Milan, Italy), according to the manufacturer’s instructions. Total RNA integrity was assessed by a 2100 Bioanalyzer (Agilent Technologies, Waldbronn, Germany). Samples with an RNA Integrity Number of at least 8.5 were used for subsequent analysis. All samples were reverse-transcribed using a GoScript™ Reverse Transcription System (Promega, Milan, Italy), according to the manufacturer’s protocol. The obtained cDNAs were used for gene expression analysis using specific 5’-3’ primer pairs designed to anneal at 56/58°C with an amplification efficiency (E) range between 2.1 and 1.9 (available on request). Real-time PCR was carried out using SsoAdvanced Universal SYBR Green Supermix (Bio-Rad, Milan, Italy) with a CFX Connect Real-Time PCR Detection System (Bio-Rad, Milan, Italy), according to the manufacturer’s instructions. Relative gene expression data were calculated using the comparative threshold cycle method (ΔΔCt) with GAPDH, μTUBULIN and SDHA as housekeeping genes.

### Statistical analysis and software

Statistical analysis was performed using InStat 5 (GraphPad, San Diego, CA, USA). Data reported are the mean±S.E.M. and were analyzed using the non-parametric Mann-Whitney test. Data expressed as percentages were analyzed using Fisher’s exact test. Only P values <0.05 were considered significant. Primer sets were designed using the Primer 3 tool; reference stability values were calculated using geNorm; and efficiency values were calculated and data analysis of the amplification runs were performed using BioRad software.

## Results

### Expression of imprinted genes and global DNA methylation in uniparental placentae

**Fig 1:**
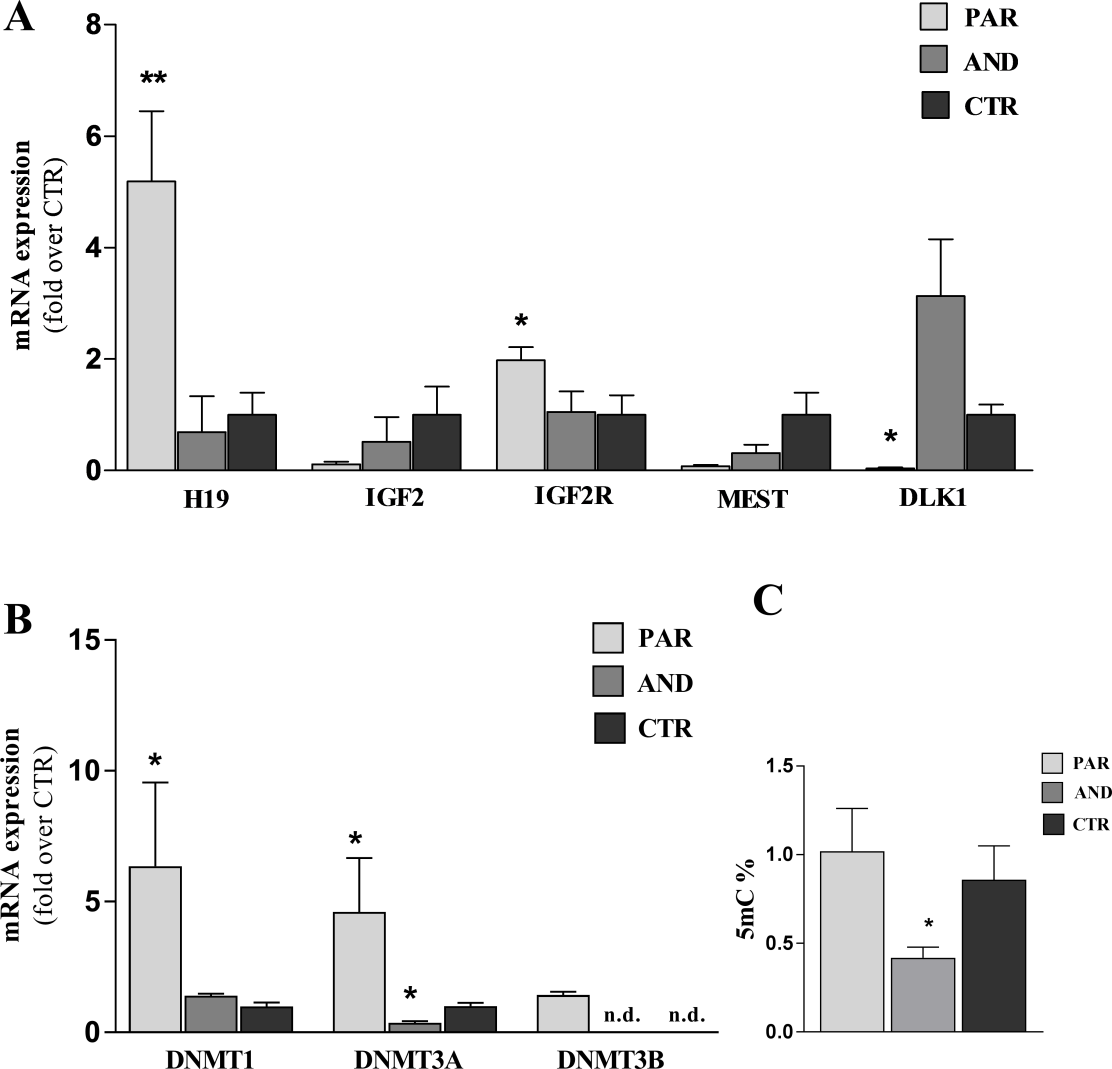
Epigenetic defects in uniparental placentae (chorion-allantoid tissues) 20 of pregnancy. A) Maternally expressed genes, *H19* and *IGF2R*, were overexpressed in PAR vs. CTR, while the paternally expressed gene *DLK1* was down regulated in PAR vs. CTR (* indicates P<0.05; ** indicates P<0.01). No significant differences were detected for *IGF2* and *MEST*. The pattern of expression was not “on” or “off” as expected, probably due to the perturbation of the epigenetic programming as a consequence of *in vitro* culture and abnormalities of our model. B) *DNMT1*, *DNMT3A* and *DNMT3B* were overexpressed in PAR vs CTR and *DNMT3A* was down expressed in AND vs CTR (* indicates P < 0.05, n.d. = not detected) C). Genome-wide methylation, assessed by 5-methylcytosine quantification, showed a significant hypomethylation in AND placentae compared to both PAR and CTR (* indicates P = 0.023).

We evaluated the expression of five imprinted genes (*DLK1*, *H19*,*IGFR2*, *IGF2* and *MEST*) and of DNA Methyltransferases (*DNMT1*, *DNMT3A* and *DNMT3B*) by qPCR. Fig 1A shows that *DLK1*, *H19* and *IGF2R* were expressed according to their parental origin. In particular, maternally expressed genes (*H19*, *IGF2R)* were overexpressed (P = 0.0079 and P = 0.0556, respectively) while paternally expressed gene (*DLK1)* was down regulated (P = 0.0286) in PAR vs. CTR. No significant differences were found for *IGF2* and *MEST*. qPCR for DNA Methyltransferases revealed overexpression of *DNMT1*, *DNMT3A* and *DNMT3B* in PAR and low expression of *DNMT3A* in AND vs CTR (P < 0.05, Fig 1B). Global DNA methylation was assessed in placental tissues at day 20 of pregnancy. The quantification of 5-mC showed hypomethylation in AND (0.42 ± 0.06), compared to both PAR (1.24 ± 0.24) and CTR (1.02 ± 0.19) (P = 0.023; Fig 1C).

### Gross morphology of uniparental placentae

**Fig 2:**
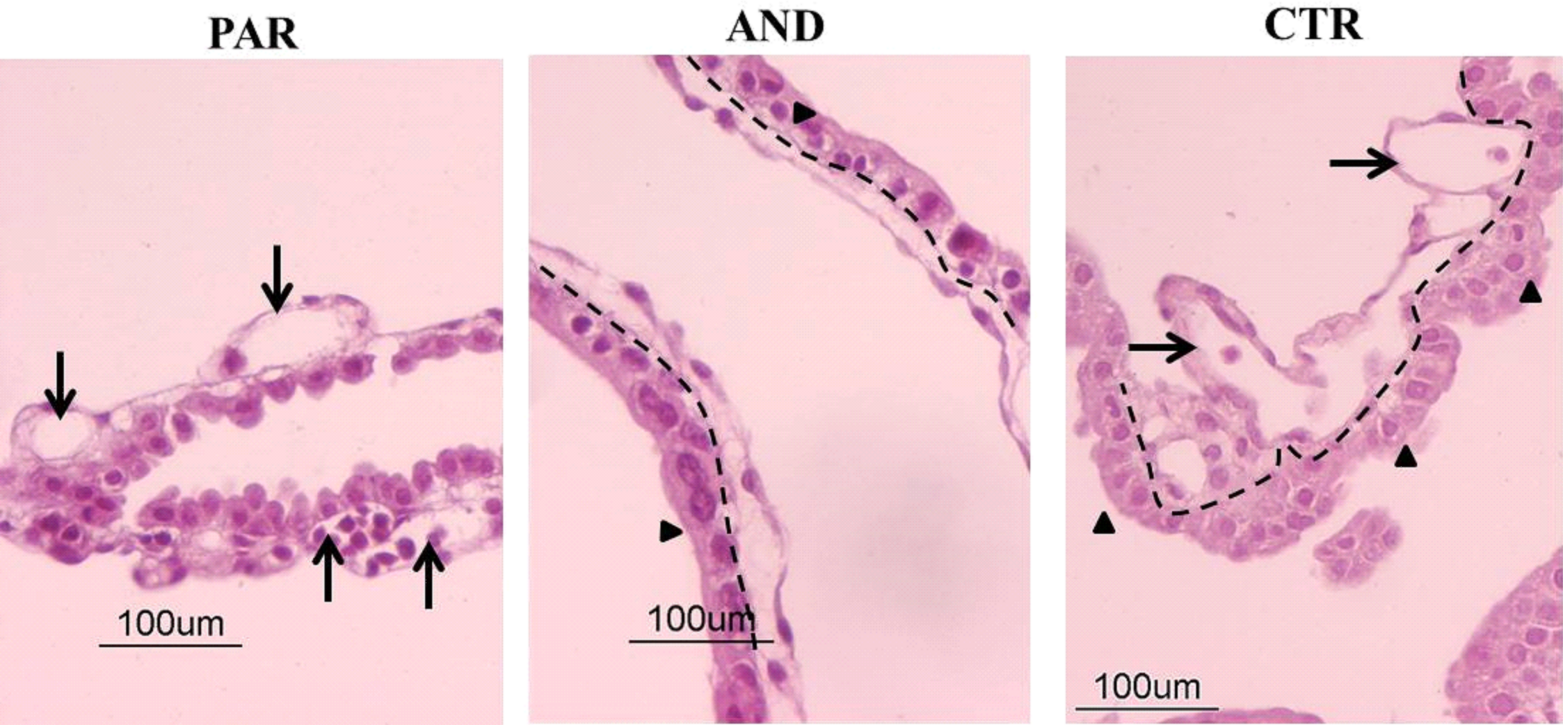
General and vascular morphology is defective in uniparental placentae (chorion-allantoid tissues) at day 20 of pregnancy. Hematoxylin & Eosin staining of uniparental (AND, PAR) chorion-allantoid tissues showed defective general morphology and vasculogenesis compared to biparental CTR, in agreement with our preliminary observations [22]. There was a well-organized trophoectodermal epithelium (arrowhead) and a well-defined basement membrane (dashed line) between chorion and allantoid in AND but not in PAR tissues. The presence of a vascular network (arrows) was observed in the PAR allantoid but not in AND.

Chorion-allantoid tissues of uniparental placentae had defective organization at day 20 of pregnancy (Fig 2). We observed a general disorganization of PAR chorion-allantoid tissues. In particular, the trophoectodermal layer lost its typical epithelial-like features and the basement membrane between chorion and allantoid was not clearly detectable compared to AND and CTR. In contrast, AND placentae were characterized by a well-organized epithelial-like trophoectodermal layer and a well-defined basement membrane between chorion and allantoid, similar to CTR. In addition, a developing vascular network was found in both PAR and CTR tissues, while it was not present in AND samples.

### Altered vasculogenesis in uniparental placentae

**Fig 3:**
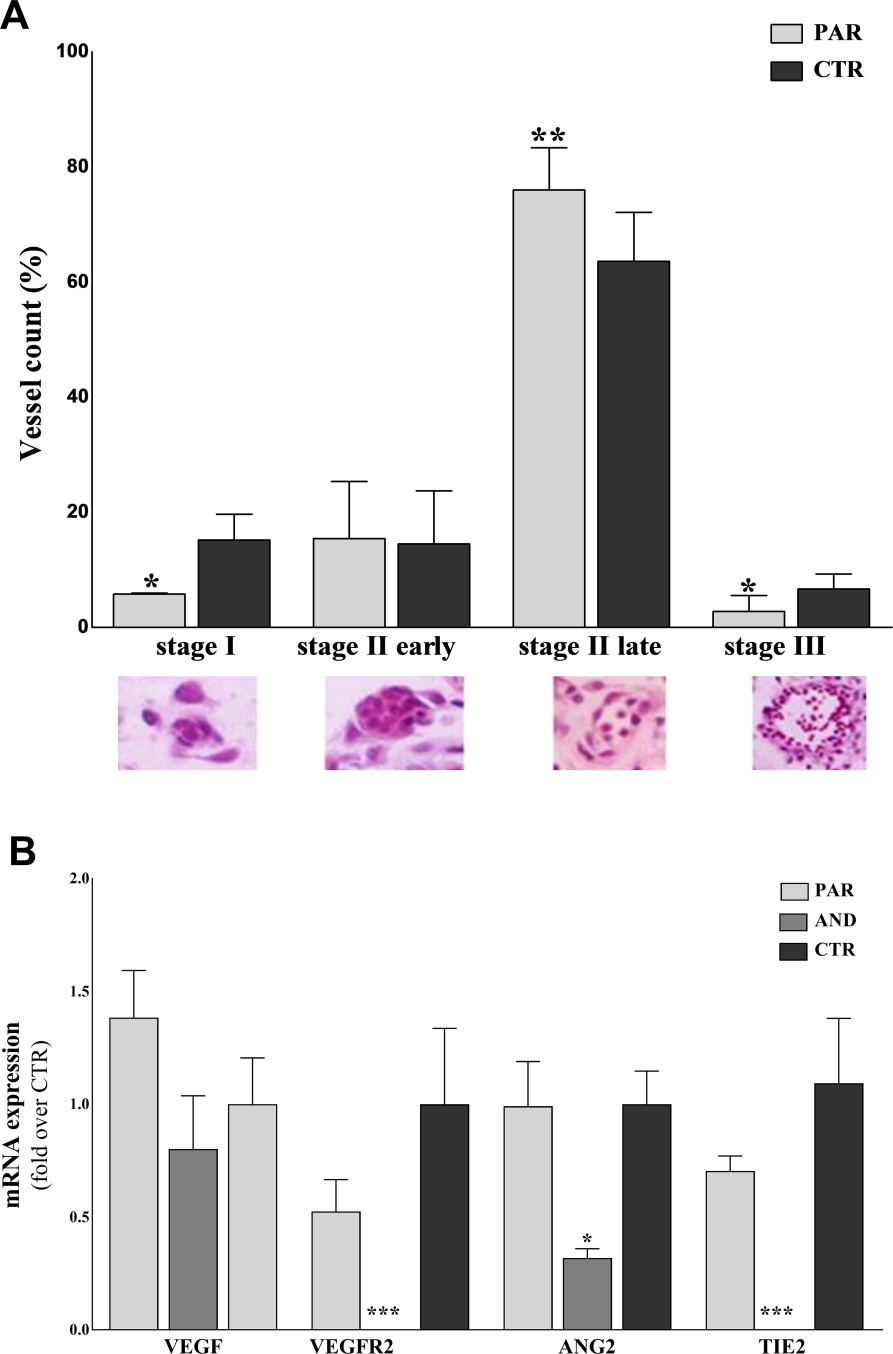
Defective vasculogenesis in uniparental placentae at day 20 of pregnancy. A) The graph shows delayed vasculogenesis in PAR placentae, with significant reduction of vessels at stages I and III and the majority of vessels at stage II late. (* indicates P<0.05, ** indicates P = 0.0066). Classification of developing vessels (*Stage I*–early vasculogenesis with formation of tight-junctional contacts between angioblasts; *Stage II early*–early tube formation with dilation of intercellular clefts and creation of the lumen precursor; *Stage II late*–development of perivascular cells resembling pericytes and hematopoietic stem cells, which pass into the early lumen; *Stage III*–late vasculogenesis/angiogenesis with establishment of a basal lamina separating the lumen and endothelial cells from the perivascular cells) was carried out only on PAR tissues, due to the lack of vessels in AND placentae (as previously observed by our group, Ptak et al, 2014). B) Reduced expression of vasculogenetic/angiogenetic factors in AND placentae at day 20 of pregnancy. The expression of genes controlling the formation and maturation of placental vessels is downregulated in AND placentae, while in PAR it is comparable to CTR (* indicates P = 0.05, *** indicates P < 0.0001).

Vasculogenesis was evaluated in placentae at 20 days of pregnancy by histological analysis (≥40 fields/sample; n ≥ 4 samples/group). Vessels were classified into four developmental stages (I, II early, II late and III; see Materials and Methods for the detailed description of each stage) (Fig 3A). We observed a delayed maturity of vessels in PAR vs. CTR. In particular, a reduced number of vessels at stages I and III and an increased number of vessels at stage II late were seen in PAR compared to the CTR (*stage I*: 5.77 ± 0.20% vs. 15.2 ± 4.48%, P = 0.0384; *stage II late* 75.99 ± 7.33% vs. 63.61 ± 8.52%, P = 0.0066; *stage III*: 2.78 ± 1.6% vs. 6.69 ± 2.60%, P = 0.044, for PAR and CTR, respectively) (Fig 3A). The analysis of vessel maturity was not performed in AND placentae, as no vessels were detected in histological sections (n ≥ 40 fields/sample, n ≥ 4 samples). (Fig 2). Then, the expression of vasculogenetic factors (*VEGF*, *VEGFR2*, *ANG2*, *TIE2*) was analyzed by qPCR (Fig 3B). Fig 3B shows a comparable expression of vasculogenetic factors between PAR and CTR, yet severe downregulation of *VEGFR2* (P<0.001) *ANG2* (P = 0.05) and *TIE2* (P<0.0001) in AND vs. CTR.

### Reduced number of Trophoblastic Binucleate Cells (BNC) in PAR placentae

**Fig 4:**
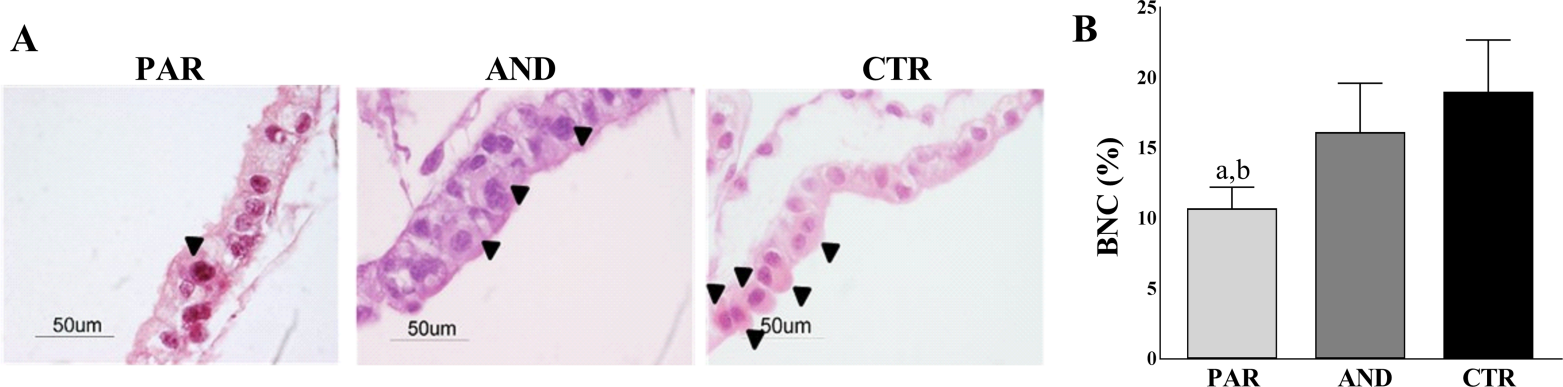
Reduced number of Trophoblastic Binucleate Cells (BNC) in PAR placentae (trophoectodermal epithelium) at day 20 of pregnancy. A) Hematoxylin & Eosin staining shows a reduced number of BNC (arrowheads) in the PAR trophoectodermal epithelium compared to AND and CTR. Note that BNC are organized in small clusters in AND and CTR but not in PAR tissues. B) The graph shows the reduced number of BNC in the PAR trophoectodermal epithelium compared to AND and CTR (*a* indicates P = 0.0248, *b* indicates P = 0.0009).

We evaluated the total number of BNC in the trophoectodermal epithelium, as they are key cells involved in the implantation process. The total number of BNC was reduced in the PAR trophoectoderm (51/479 cells, 10.73 ± 1.15%) vs. both CTR (78/432 cells, 19.03 ± 3.67%, *a* denotes P = 0.0009) and AND (61/383, 16.14 ± 3.47%, *b* denotes P = 0.0248) (Fig 4A and 4B).

### Reduced survival rate in uniparental embryos

**Fig 5:**
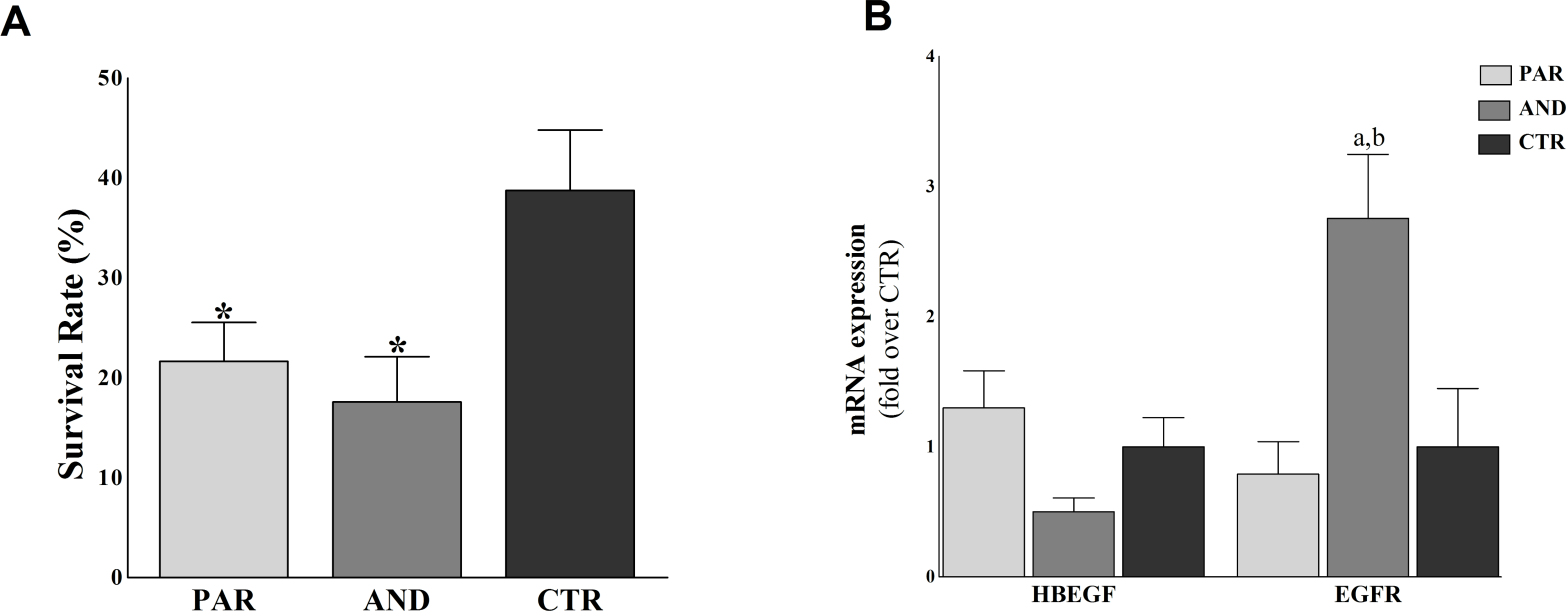
Impaired developmental competence of uniparental conceptuses. A) Reduced survival rates of uniparental embryos (AND, PAR) compared to biparental CTR ones. Survival rate was calculated as number of collected conceptuses/number of transferred embryos. (* indicates P<0.03 vs. CTR). B) Up-regulation of *EGFR* in AND vs. CTR and PAR placentae. (* denotes P = 0.03 vs CTR; ** denotes P = 0.017 vs PAR).

Reduced survival rate was found in PAR (13/60, 21.68 ± 3.86%, P = 0.0287) and AND (9/60, 17.59 ± 4.2%, P = 0.0179) vs. CTR (11/30, 38.78 ± 5.87%) (Fig 5A). We also analyzed the expression of *HBEGF* and its receptor *EGFR* by qPCR, as they are main actors of feto-maternal communication during implantation. Fig 5B shows increased expression of *EGFR* in AND vs. CTR (P = 0.03) and PAR (P = 0.017). Comparable expression was observed for *HBEGF* among all groups (P>0.05).

## Discussion

The present study provides new insight in the characterization of early placentation in uniparental models (PAR–only maternal genome- and AND- only paternal genome). In particular, our results show that the paternal genome isinvolved in the development of the trophoectodermal layer and the maternal genome is involved in vasculogenesis.

The survival rate of both PAR and AND was reduced compared to CTR, as shown previously in mouse [2], cow [23] and sheep [22]. Imprinted genes *H19*, *IGF2R* (maternally-expressed) and *DLK1* (paternally-expressed) showed an expression profile according to their parental origin while no significant differences were observed for *IGF2 and MEST*. In this study, the pattern of expression of imprinted genes was not “on” or “off” as would be expected due to their parental origin. Similarly, disruption of parental-specific expression of imprinted genes has been observed also in mouse uniparental cell lines [24] and fetuses [25].This result could be explained as effect of 1) the highly abnormality of uniparental tissues (in particular AND) or 2) *in vitro* culture, as it is responsible of increased frequency of stochastic epigenetic errors at imprinted genes in placental tissues [26]. Moreover, it has been reported that the introduction of epigenetic marks can be reinforced/completed even after blastocyst stage [27,28], thus it can be speculated that at 20 days of pregnancy the monoallelic expression of the analyzed imprinted genes is not yet fully established in our model. The disruption of parental-specific expression of imprinted genes could also be due to the observed dysregulation of *DNMTs*, key genes in the establishment and maintenance of DNA methylation, especially in PAR placentae. This observation is in agreement with our and other previous studies [13,29], where the impaired expression of *DNMTs* as well as imprinted genes in ovine placentae has been associated with ART protocols (*i*.*e*. superovulation, *in vitro* culture) and/or embryo origin (cloned and parthenogenetic tissues). Global DNA methylation analysis revealed hypomethylation of AND placentae vs CTR. This finding differs somehow from our previous study [19], where we reported increased methylation status in PAR blastocysts. However, we should consider that both analysis were performed on two different developmental stages (blastocyst vs. early placentae) using two different methods (immunofluorescence–semiquantitative analysis- vs.ELISA–quantitative analysis). Moreover, evaluation of methylation on blastocyst was carried out on whole embryos (both inner cell mass and trophectoderm) while in the present study we analyzed only placental tissues (originating from trophectoderm) but not fetuses.

Morphological observation of uniparental placental tissues showed a well-organized epithelial-like trophoectodermal layer and a well-defined basement membrane between chorion and allantois in AND, whereas altered organization of the trophoectodermal layer and a significant reduction of BNC in PAR was observed. Similar deficits have been observed in mouse PAR, where a low number of Trophoblastic Giant Cells (TGC, analogous to BNC) was correlated to the absence/reduction of trophoblastic stem cells [24,30,31]. However, ovine BNC originate from acytokinetic mitosis of uninucleate trophoblastic cells, not from stem cells [32], thus the comparison of the two models presents some limitation. Studies aimed to better characterize this sub-population of trophoblastic cells should be performed in the sheep model to confirm/deny species-specific origin of BNC.

As trophectoderm development plays a crucial role in the establishment of pregnancy, we evaluated *HBEGF* and its receptor *EGFR* expression, as they are main actors in feto-maternal communication during implantation. Our data showed an overexpression of *EGFR* in AND placentae compared to both CTR and PAR. As *EGFR* promotes the proliferative and invasive properties of trophoblasts [33], its overexpression associated with the proper organization of the trophoectodermal layer can suggest a higher invasive potential of AND placentae, resembling what is generally observed in hydatidiform moles.

We found delayed vasculogenesis in PAR placentae, while no signs of vasculogenesis were detected in analyzed AND tissues (n ≥40 fields/samples; n ≥40 sample/group). These observations were further confirmed by the expression of the selected panel of vasculogenetic factors (*VEGF*, *VEGFR2*, *ANG2*, *TIE2*). PAR and CTR showed a comparable expression profile of these factors, while a severe downregulation of *VEGFR2*, *ANG2* and *TIE2* was found in AND. The defective vascular network in AND can be correlated to impaired feto-maternal communication, especially to a reduced transfer of nutrients [20]. The severe vasculogenetic defect may also explain the increased level of autophagy in AND placentae [22]. In fact, autophagy is a cellular mechanism that may be activated to compensate the reduced intake of nutrients to the fetus [22]. Even if the increased autophagy may be a compensatory mechanism to support fetal growth, the defective vasculogenesis is not completely in agreement with the role of paternally expressed genes as key elements in regulating nutrient supply to promote fetal development, as mainly described in mouse model [34]. This discrepancy could be explained by species-specific differences between sheep and mouse (*i*.*e*., different placentation and timing of implantation).

Our study provides new insight in the characterization of early placentation of parthenogenetic (only-maternal genome) and androgenetic (only-paternal genome) conceptuses and suggests that the maternal genome drives vasculogenesis, while the paternal genome is important for trophectoderm development. As the successful establishment of pregnancy requires the proper development of both vascular network (for nutrient supply) and trophectoderm (for fetal-maternal interaction), our results contribute to a better understanding of the etiology of placental diseases (*i*.*e*. placenta accreta, intra uterine growth restriction) and to the development of new therapeutic strategies for their diagnosis and treatment. Further investigations are necessary to confirm our results and to identify the main mechanisms involved in defective placental development, which could be correlated with placental insufficiency and pregnancy loss/complications.

## References

[1] McGrath J, Solter D. Completion of mouse embryogenesis requires both the maternal and paternal genomes. Cell. 1984;37: 179–183. doi: 10.1016/0092-8674(84)90313-1 6722870 10.1016/0092-8674(84)90313-1

[2] Surani MAH, Barton SC, Norris ML. Development of reconstituted mouse eggs suggests imprinting of the genome during gametogenesis. Nature. 1984;308: 548–550. doi: 10.1038/308548a0 6709062 10.1038/308548a0

[3] Surani MAH, Barton SC, Norris ML. Nuclear transplantation in the mouse: Heritable differences between parental genomes after activation of the embryonic genome. Cell. 1986;45: 127–136. doi: 10.1016/0092-8674(86)90544-1 3955655 10.1016/0092-8674(86)90544-1

[4] Constância M, Kelsey G, Reik W. Resourceful imprinting. Nature. 2004;432: 53–7. doi: 10.1038/432053a 15525980 10.1038/432053a

[5] Coan PM, Burton GJ, Ferguson-Smith AC. Imprinted genes in the placenta—a review. Placenta. 2005;26 Suppl A: S10–20. doi: 10.1016/j.placenta.2004.12.009 15837057 10.1016/j.placenta.2004.12.009

[6] Gutierrez-Marcos JF, Constância M, Burton GJ. Maternal to offspring resource allocation in plants and mammals. Placenta. 2012;33 Suppl 2: e3–10. doi: 10.1016/j.placenta.2012.08.006 22995735 10.1016/j.placenta.2012.08.006

[7] Frost JM, Moore GE. The importance of imprinting in the human placenta. PLoS Genet. Public Library of Science; 2010;6: e10001015 doi: 10.1371/journal.pgen.1001015 20617174 10.1371/journal.pgen.1001015 PMC2895656

[8] Bressan FF, De Bem THC, Perecin F, Lopes FL, Ambrosio CE, Meirelles F V, et al Unearthing the roles of imprinted genes in the placenta. Placenta. Elsevier Ltd; 2009;30: 823–34. doi: 10.1016/j.placenta.2009.07.007 19679348 10.1016/j.placenta.2009.07.007

[9] Angiolini E, Coan PM, Sandovici I, Iwajomo OH, Peck G, Burton GJ, et al Developmental adaptations to increased fetal nutrient demand in mouse genetic models of Igf2-mediated overgrowth. FASEB J. 2011;25: 1737–1745. doi: 10.1096/fj.10-175273 21282203 10.1096/fj.10-175273

[10] Fowden AL, Sibley C, Reik W, Constancia M. Imprinted genes, placental development and fetal growth. Horm Res. 2006;65: 50–58. doi: 10.1159/000091506 16612114 10.1159/000091506

[11] Leighton P, A., Ingram R, S., Eggenschwiler J, Efstratiadis A, et al Disruption of imprinting caused by deletion of the H19 gene region in mice. Nature. 1995;375: 34–39. doi: 10.1038/375034a0 7536897 10.1038/375034a0

[12] Nelissen ECM, Dumoulin JCM, Busato F, Ponger L, Eijssen LM, Evers JLH, et al Altered gene expression in human placentas after IVF/ICSI. Hum Reprod. 2014;29: 2821–31. doi: 10.1093/humrep/deu241 25316457 10.1093/humrep/deu241

[13] Ptak GE, D’Agostino A, Toschi P, Fidanza A, Zacchini F, Czernik M, et al Post-implantation mortality of in vitro produced embryos is associated with DNA methyltransferase 1 dysfunction in sheep placenta. Hum Reprod. 2013;28: 298–305. doi: 10.1093/humrep/des397 23169866 10.1093/humrep/des397

[14] Diplas AI, Lambertini L, Lee MJ, Sperling R, Lee YL, Wetmur J, et al Differential expression of imprinted genes in normal and IUGR human placentas. Epigenetics. 2009;4: 235–240. 9019 [pii] 19483473 10.4161/epi.9019

[15] Yu L, Chen M, Zhao D, Yi P, Lu L, Han J, et al The H19 gene imprinting in normal pregnancy and pre-eclampsia. Placenta. 2009;30: 443–7. doi: 10.1016/j.placenta.2009.02.011 19342096 10.1016/j.placenta.2009.02.011

[16] Barry JS, Anthony R V. The pregnant sheep as a model for human pregnancy. Theriogenology. 2008;69: 55–67. doi: 10.1016/j.theriogenology.2007.09.021 17976713 10.1016/j.theriogenology.2007.09.021 PMC2262949

[17] Barry JS, Rozance PJ, Anthony R V. An animal model of placental insufficiency-induced intrauterine growth restriction. Semin Perinatol. 2008;32: 225–30. doi: 10.1053/j.semperi.2007.11.004 18482626 10.1053/j.semperi.2007.11.004

[18] Ptak GE, Clinton M, Tischner M, Barboni B, Mattioli M, Loi P. Improving Delivery and Offspring Viability of In Vitro-Produced and Cloned Sheep Embryos. Biol Reprod. 2002;67: 1719–1725. doi: 10.1095/biolreprod.102.006171 12444045 10.1095/biolreprod.102.006171

[19] Zacchini F, Czernik M, Iuso D, Toschi P, di Egidio F, Scapolo PA, et al Efficient production and cellular characterization of sheep androgenetic embryos. Cell Reprogram. 2011;13: 495–502. doi: 10.1089/cell.2011.0021 22043807 10.1089/cell.2011.0021 PMC3229817

[20] Charnock-Jones DS, Kaufmann P, Mayhew TM. Aspects of human fetoplacental vasculogenesis and angiogenesis. I. Molecular regulation. Placenta. 2004;25: 103–13. doi: 10.1016/j.placenta.2003.10.004 14972443 10.1016/j.placenta.2003.10.004

[21] Demir R, Seval Y, Huppertz B. Vasculogenesis and angiogenesis in the early human placenta. Acta Histochem. 2007;109: 257–65. doi: 10.1016/j.acthis.2007.02.008 17574656 10.1016/j.acthis.2007.02.008

[22] Ptak GE, Toschi P, Fidanza A, Czernik M, Zacchini F, Modlinski JA, et al Autophagy and apoptosis: parent-of-origin genome-dependent mechanisms of cellular self-destruction. Open Biol. 2014;4: 140027 doi: 10.1098/rsob.140027 24898141 10.1098/rsob.140027 PMC4077060

[23] Lagutina I, Lazzari G, Duchi R, Galli C. Developmental Potential of Bovine Androgenetic and Parthenogenetic Embryos: A Comparative Study. Biol Reprod. 2004;70: 400–405. doi: 10.1095/biolreprod.103.021972 14561645 10.1095/biolreprod.103.021972

[24] Ogawa H, Shindo N, Kumagai T, Usami Y, Shikanai M, Jonwn K, et al Developmental ability of trophoblast stem cells in uniparental mouse embryos. Placenta. 2009;30: 448–56. doi: 10.1016/j.placenta.2009.02.006 19345411 10.1016/j.placenta.2009.02.006

[25] Ogawa H, Wu Q, Komiyama J, Obata Y, Kono T. Disruption of parental-specific expression of imprinted genes in uniparental fetuses. FEBS Lett. 2006;580: 5377–5384. doi: 10.1016/j.febslet.2006.08.087 16987518 10.1016/j.febslet.2006.08.087

[26] de Waal E, Mak W, Calhoun S, Stein P, Ord T, Krapp C, et al In Vitro Culture Increases the Frequency of Stochastic Epigenetic Errors at Imprinted Genes in Placental Tissues from Mouse Concepti Produced Through Assisted Reproductive Technologies. Biol Reprod. 2014;90: 22–22. doi: 10.1095/biolreprod.113.114785 24337315 10.1095/biolreprod.113.114785 PMC4076403

[27] Srivastava M, Frolova E, Rottinghaus B, Boe SP, Grinberg A, Lee E, et al Imprint control element-mediated secondary methylation imprints at the Igf2/H19 locus. J Biol Chem. American Society for Biochemistry and Molecular Biology; 2003;278: 5977–83. doi: 10.1074/jbc.M208437200 12270940 10.1074/jbc.M208437200

[28] Thurston A, Taylor J, Gardner J, Sinclair KD, Young LE. Monoallelic expression of nine imprinted genes in the sheep embryo occurs after the blastocyst stage. Reproduction. Society for Reproduction and Fertility; 2008;135: 29–40. doi: 10.1530/REP-07-0211 18159081 10.1530/REP-07-0211

[29] Grazul-Bilska AT, Johnson ML, Borowicz PP, Baranko L, Redmer DA, Reynolds LP. Placental development during early pregnancy in sheep: Effects of embryo origin on fetal and placental growth and global methylation. Theriogenology. 2013;79: 94–102. doi: 10.1016/j.theriogenology.2012.09.013 23117132 10.1016/j.theriogenology.2012.09.013 PMC3518681

[30] Hoffman LH, Wooding FB. Giant and binucleate trophoblast cells of mammals. J Exp Zool. 1993;266: 559–577. doi: 10.1002/jez.1402660607 8371098 10.1002/jez.1402660607

[31] Newman-Smith E, Werb Z. Functional analysis of trophoblast giant cells in parthenogenetic mouse embryos. Dev Genet. 1997;20: 1–10. doi: 10.1002/(SICI)1520-6408(1997)20:1<1::AID-DVG1>3.0.CO;2-B 9094206 10.1002/(SICI)1520-6408(1997)20:1<1::AID-DVG1>3.0.CO;2-B

[32] Wimsatt WA. Observations on the morphogenesis, cytochemistry, and significance of the binocleate giant cells of the placenta of ruminants. Am J Anat. 1951;89: 233–81. doi: 10.1002/aja.1000890204 14894441 10.1002/aja.1000890204

[33] Mochizuki M. Trophoblast: its functional regulation and pathophysiological profiles. Nihon Sanka Fujinka Gakkai Zasshi. 1992;44: 918–28. Available: http://www.ncbi.nlm.nih.gov/pubmed/1402224 1402224

[34] Moore T, Haig D. Genomic imprinting in mammalian development: a parental tug-of-war. Trends Genet. 1991;7: 45–49. doi: 10.1016/0168-9525(91)90230-N 2035190 10.1016/0168-9525(91)90230-N

